# Clinical Features of Patients with Exfoliation Glaucoma Requiring Surgical Intervention

**DOI:** 10.1155/2020/9423756

**Published:** 2020-06-19

**Authors:** Hideyuki Nakano, Tetsuya Togano, Yuta Sakaue, Aki Suetaka, Ryu Iikawa, Rieko Nakano, Takeo Fukuchi

**Affiliations:** Department of Ophthalmology, Niigata University Graduate School of Medical and Dental Science, 1-757 Asahimachi-dori, Chuo-ku, Niigata 951-8510, Japan

## Abstract

**Purpose:**

To clarify the clinical features of patients with exfoliation glaucoma (XFG) requiring surgical intervention. *Study Design*. Retrospective study.

**Methods:**

The study included 46 eyes from 36 XFG patients, 85 eyes from 53 primary open-angle glaucoma (POAG) patients, and 54 eyes from 35 normal-tension glaucoma (NTG) patients. Age, duration of previous glaucoma treatment, intraocular pressure, medication scores, visual function, and surgical procedure were compared among the three patient groups.

**Results:**

The XFG group had the highest mean age (XFG: 75.7 ± 8.3 years, POAG: 65.8 ± 12.8 years, and NTG: 53.3 ± 12.8 years; *p* < 0.001) and the shortest mean duration of previous treatment with glaucoma medication (XFG: 5.1 ± 3.5 years, POAG: 8.9 ± 6.9 years, and NTG: 8.9 ± 5.9 years; *p* < 0.001). Intraocular pressure and medication scores were slightly higher in the XFG group than in the POAG group, although the differences were not significant. Among XFG patients, trabeculectomy was performed in 20 eyes from 16 patients (55.6%) and trabeculotomy was performed in 16 eyes from 14 patients (44.4%). Both trabeculectomy (3 eyes) and trabeculotomy (14 eyes) were performed in combination with cataract surgery.

**Conclusions:**

The XFG patients referred to our department for initial examination were older than the POAG and NTG patients, and their duration of treatment before referral was shorter. Moreover, intraocular pressure and the eye drop medication score were higher in the XFG patients. A significantly higher percentage of XFG patients required surgical intervention compared to patients with other disease types.

## 1. Introduction

Exfoliation glaucoma (XFG) is a secondary open-angle glaucoma that occurs in one or both eyes, mainly among the elderly. This secondary glaucoma is most prevalent in patients aged ≥70 years, and it is the most frequent open-angle glaucoma associated with high intraocular pressure (IOP) in this age group [1–4]. A previous multicenter study found that XFG was present in 40–60% of patients with exfoliation syndrome (XFS), with a prevalence of 0.2% among those aged ≥40 years and 1.2% among those aged ≥80 years [5]. Although XFG is a type of secondary glaucoma, due to the current lack of causal therapy, it is treated similarly to primary open-angle glaucoma (POAG). For example, in clinical research in the United States and Europe, XFG, such as POAG and normal-tension glaucoma (NTG), is often treated as open-angle glaucoma [6, 7]. Thus, drugs are initially administered, while surgical treatment is considered if adjustment of IOP is difficult or if there is progressive visual field impairment [8]. The clinical features of XFG patients differ from those of POAG and NTG patients; XFG patients are older at diagnosis, have more severe visual field constriction, have higher IOP, and exhibit greater variability [9–16]. In addition, since pharmacotherapy for glaucoma is usually started in elderly patients, and the visual function of the unaffected eye is often relatively good, patients themselves often do not realize the seriousness of the condition. Thus, it is not uncommon for adherence to be problematic and for adequate management of IOP to be infeasible, resulting in rapid progression and remarkably impaired residual visual function. Futa has reported that visual dysfunction often worsens in situations where the IOP can be adjusted; these include instances in which the treatment periods can be set, for example, if pharmacotherapy is initiated in accordance with POAG treatment, and surgical treatment is planned if the effect is inadequate [17]. Considering the various clinical features of XFG described above, treatment and management plans that differ from those for POAG and NTG should be adopted in XFG patients.

Therefore, this study investigated the clinical features of XFG patients who were referred to our department or who visited our institution and compared XFG patients who were candidates for surgical treatment with POAG and NTG patients. The treatment policy and management of XFG patients were reconsidered based on the results.

## 2. Materials and Methods

This study included 185 eyes from 124 of the 313 glaucoma patients who met the following criteria and who first visited the Department of Ophthalmology, Niigata University Medical and Dental Hospital, between April 2013 and March 2014. The inclusion criteria were age ≥20 years; diagnosis of XFG, POAG, or NTG; and the need for glaucoma surgery at the time of referral or thereafter. The exclusion criteria were a history of surgery for glaucoma, macular disease, corneal disease, intracranial disease, or diseases other than glaucoma that might affect visual acuity and visual field and failure to consent to the use of clinical information by this study. This retrospective study was conducted in accordance with the Helsinki Declaration of the World Medical Association and was approved for publication by the Investigation Review Board of Niigata University.

All new patients who presented to Niigata University Medical and Dental Hospital as glaucoma outpatients had already been diagnosed with some form of glaucoma or had been referred due to suspected glaucoma by related ophthalmic clinics/institutions. At the time of consultation at our department, according to usual ophthalmologic practice, patients underwent visual acuity assessment, refraction assessment, IOP assessment (via Goldmann applanation tonometer), slit-lamp examination, angle examination, dilated pupil ophthalmoscopy and fundoscopy, ocular fundus photography, optic nerve head and macular analysis via optical coherence tomography (3D-OCT 2000; Topcon, Tokyo, Japan), and geometric analysis of the anterior eye segment via optical coherence tomography (CASIA; Tomey, Tokyo, Japan). The glaucoma type was diagnosed and classified according to the 3rd edition of the Guidelines for Glaucoma Eye Care. Individuals were classified as XFG patients if one eye had XFG and the other had POAG or NTG and were classified as POAG patients if one eye had POAG and the other had NTG. All IOP values were measured using a Goldmann applanation tonometer. Visual field examinations included dynamic quantitative visual field measurements using a Goldmann applanation tonometer with Humphrey visual field analyzer (HFA; Carl Zeiss Meditec, Dublin, CA, USA) with 30/24-2 programs and Swedish Interactive Thresholding Algorithm standards. The presence or absence of visual field abnormalities due to glaucoma was assessed in accordance with the criteria proposed by Anderson and Patella [18].

In this study, the medical data provided by the referring institutions and the examination results during visits to our institution were used to determine the disease type (XFG, POAG, or NTG), ages at glaucoma diagnosis and treatment initiation, duration between treatment initiation and referral to our institution, IOP at treatment initiation, age at the visit to our institution, IOP values, scores for drugs used (single-agent eye drops, 1 point; combination eye drops, 2 points; and each 250 mg tablet of the carbonic anhydrase inhibitor acetazolamide, 1 point), and degree of visual field disorder. Mean deviation (MD) and visual field index (VFI) values were used as indicators of visual field impairment. Patients who did or did not require surgery were compared in terms of disease type, and XFG patients were compared according to the surgical procedure. Surgical indications were determined by the same group of clinicians specializing in glaucoma based on uniform criteria. In particular, patients were considered eligible for surgery if IOP was elevated or if visual field disturbance had advanced beyond an acceptable range. Trabeculectomy was performed as filtration surgery with or without ExPRESS®, and trabeculotomy (extraocular or intraocular) was performed as an aqueous outflow tract surgery. Both approaches involved an isolated surgery and a surgery in combination with cataract surgery. When only cataract surgery was performed, patients were considered not to have undergone glaucoma surgery.

For statistical analyses, the Tukey–Kramer method was used as a multiple comparison test for comparisons of means in each group, and the chi-square test was used for comparisons of sample sizes in each group. All analyses were performed using IBM SPSS Statistic Version 25 (IBM Corp., Armonk, NY, USA).

## 3. Results

Among 313 patients who visited our institution during the study period, 74 (23.6%) were diagnosed with POAG, 55 (17.6%) were diagnosed with NTG, 48 (15.3%) were diagnosed with XFG, and 136 were diagnosed with other conditions. Among these patients, 124 were included in this study. According to disease type, there were 46 eyes from 36 XFG patients, 85 eyes from 53 POAG patients, and 54 eyes from 35 NTG patients. The following details are provided in Table 1: age at the initial visit to our institution, sex, duration of treatment, IOP value, eye drop instillation score, and visual field score (MD and VFI values).

**Figure 1:**
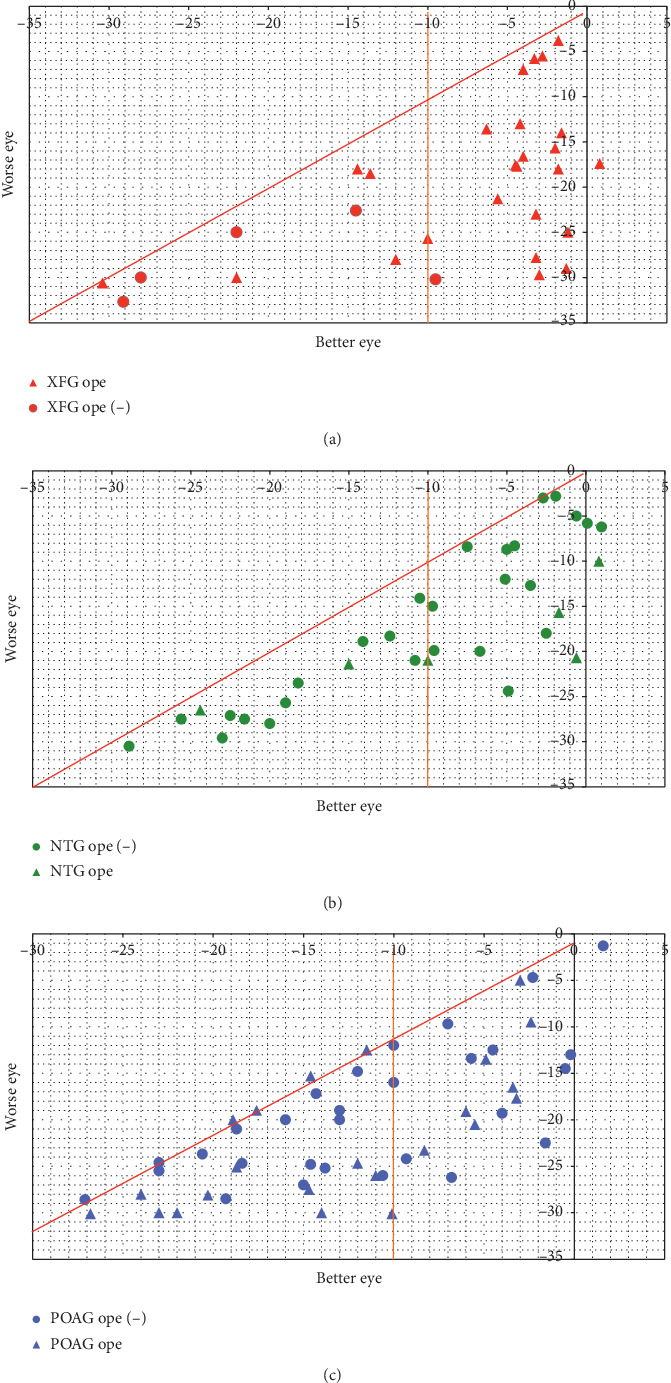
(a) Scatter plot of the Humphrey visual field 24-2 or 30-2 program and Swedish Interactive Thresholding Algorithm standard values for the better eye and worse eye, with mean deviation values of XFG and indication for surgery (yes/no). (b). Scatter plot of the Humphrey visual field 24-2 or 30-2 program and Swedish Interactive Thresholding Algorithm standard values for the better eye and worse eye, with mean deviation values of NTG and indication for surgery (yes/no). (c). Scatter plot of the Humphrey visual field 24-2 or 30-2 program and Swedish Interactive Thresholding Algorithm standard values for the better eye and worse eye, with mean deviation values of POAG and indication for surgery (yes/no).

Overall, 34 eyes (74.4%) from 31 XFG patients, 28 eyes (33.7%) from 23 POAG patients, and 6 eyes (11.1%) from 6 NTG patients were considered candidates for surgical treatment; the rate was significantly higher among XFG patients than among POAG and NTG patients (Table 2; *p* < 0.001). When limited to patients aged ≥80 years, 14 of 24 eyes from XFG patients, 4 of 12 eyes from POAG patients, and 0 of 5 eyes from NTG patients were considered candidates for surgical treatment (Table 2; XFG vs. POAG; *p*=0.039). The mean age, IOP values, drug scores, and visual field scores of patients who were considered to be surgical candidates are shown in Table 3. Scatter plots of eyes, with higher (better eyes) and lower (worse eyes) MD values according to the indication for surgery (yes/no), are shown in Figures 1(a)–1(c).  Table 4 shows the number of eyes, age, duration until referral to our institution, IOP values at the visit to our institution, eye drop instillation scores, and visual field scores for eyes with glaucoma according to the surgical procedure of filtering surgery and aqueous outflow tract surgery among eyes for which glaucoma surgery is or is not indicated. In patients considered to be surgical candidates, 21 (80.8%) of the 26 XFG eyes, 10 (43.5%) of the 23 POAG eyes, and 4 (66.7%) of the 6 NTG eyes (Table 5; XGF vs. POAG; *p*=0.025) showed MD values for the better eye that were greater than or equal to −10 dB.

## 4. Discussion

This study evaluated the clinical features of XFG patients who were referred to and who presented to our institution and compared their findings with those of POAG and NTG patients. XFG patients were significantly older at diagnosis, had a significantly shorter treatment duration until referral, and were significantly older at presentation to our institution when compared with POAG and NTG patients. Among the three patient groups, XFG patients showed the highest eye drop instillation scores, the highest IOP values, and the most advanced visual field impairment. In addition, among the referred patients, the XFG group had the highest proportion of patients considered to be surgical candidates. In particular, the proportion was the highest in patients aged ≥80 years, and this group had the largest number of patients. These findings demonstrate that XFG is the most common type of high-IOP glaucoma in older patients, and it is a disease in which pharmacotherapy requires a large number of drug combinations; however, high IOP and visual field impairment remain difficult to control, and both have recently been commonly described as features of XFG. Our results demonstrated that numerous patients were considered to be surgical candidates and that an overwhelmingly large number of patients requiring glaucoma surgery were aged ≥80 years.

When the evaluation was limited to surgical patients, the mean age, IOP values, and ocular and oral medication scores were significantly higher in XFG patients than in POAG and NTG patients. Additionally, both MD values (derived with HFA) and the VFI were highest in XFG patients. While trends in age and IOP values were similar in surgical patients to those in all referred patients, visual field impairment was rather mild in the former group. These findings indicate that while the indication for surgery in POAG and NTG patients is mainly determined by the degree of visual field impairment rather than by high IOP, XFG patients present with a relatively high IOP that cannot be controlled, leading to the need for surgery in many patients. In addition, the glaucoma surgery performed in all NTG patients and most POAG patients was trabeculectomy (data not shown). By contrast, trabeculotomy was performed in fewer than 40% of the XFG patients. Similar to steroid-induced glaucoma, XFG is an appropriate indication for trabeculotomy [19]. This surgical procedure is less invasive and less burdensome for both the patient and the surgeon when compared with trabeculectomy. Thus, older XFG patients present with conditions that make it easy to select trabeculotomy, and this is considered to have greatly affected the present results. For example, considering the surgical procedure selected for XFG patients, the MD and VFI values of XFG patients who elected to undergo filtering surgery were clearly lower than the values of POAG and NTG patients (Table 3).

Scatter plots for better and worse eyes in terms of MD values were created according to disease type and surgery implementation (Figures 1(a)–1(c)). Significantly more XFG patients showed MD values of the better eye of greater than or equal to −10 dB when compared with POAG and NTG patients. XFG is believed to be a systemic disease that is likely to eventually become bilateral [1–4]. Clinically, however, patients in whom both eyes develop XFG almost simultaneously are rare. Thus, at diagnosis, the affected eye typically presents with advanced visual field impairment, whereas the other eye typically presents with no or mild visual field impairment. This presentation is considered to be one of the clinical features of XFG. Therefore, the patient's quality of life is considered to be relatively good at the time of treatment initiation. This is one of the key points to be considered when selecting XFG treatment and management.

With the increased life span and aging of the Japanese population, different perspectives and problems have arisen regarding the treatment and control of glaucoma. Adherence might be the greatest barrier to pharmacotherapy for glaucoma. As adherence often declines with advancing age [20], there are many patients in whom the treatment and control of glaucoma become unstable. The treatment of XFG, a condition that develops as patients become older and often presents with high IOP requiring therapy with multiple medications, is likely to become a significant issue in the future. It is necessary to understand that XFG patients present with a variety of conditions that make it difficult to improve or maintain adherence. In XFG patients, if the progression of IOP and visual field impairment is difficult to control, the early introduction of surgical treatment might reduce the burden on these patients and improve visual function prognosis. In particular, XFG is treated well with trabeculotomy, and cataract surgery might further enhance and stabilize the observed effects [19, 21, 22]. Given the mechanism of IOP elevation observed in XFG, the early introduction of aqueous outflow tract surgery might be advisable. Thus, although the mechanism of elevated IOP in XFG has not been elucidated in detail, the deposition of desquamating material in the trabeculae and the reduction in trabecular function are considered to be key factors. Pathologically, in the early stage of XFG, deposition of desquamative material in the trabecular meshwork is observed, and Schlemm's canal is opened; however, as the disease progresses, the trabecular space is blocked, resulting in further narrowing and occlusion of Schlemm's canal and the collecting duct [3]. This suggests that in XFG patients, the effect of aqueous outflow tract surgery with regard to opening the trabeculae is more likely to occur before the condition becomes severe. Minimally invasive glaucoma surgery has recently attracted attention [23]. The intraocular trabeculotomy method is considered to be such an approach; the procedure is technically easier than the conventional extraocular method, the surgical duration is short, and there is obviously minimal invasion.

One of the limitations of this study is the fact that the study population included glaucoma patients who were referred to our institution, and thus, the research was hospital-based. The glaucoma patients who presented to our institution as outpatients were limited to referred patients. In addition, there were no set criteria for the type of glaucoma, disease stage, treatment status, or referral purpose at the time of referral, and assessments were made by the referring ophthalmologist. Furthermore, the glaucoma patients seen at our institution lived not only in Niigata Prefecture but also in neighboring Yamagata and Fukushima Prefectures; this broad area may be associated with differences in medical care, and thus, our results may have been influenced by the treatment preferences of local ophthalmologists. Although elderly XFG patients in this study comprised the majority of patients who required glaucoma surgery, it was unclear how many patients diagnosed with XFG required surgical treatment. In addition, this study focused on analyses regarding the implementation or nonimplementation of surgery. In fact, 2 eyes from 2 patients who were considered to be candidates for glaucoma surgery did not undergo surgery, as the patients, supported by their families, did not want surgical treatment. It is necessary to consider the fact that the decision to perform surgery takes into account the balance between efficacy and side effects, and as such, the indications for filtration and aqueous outflow tract surgeries cannot be treated equally.

There exists a deeply rooted opinion that adequate lowering of IOP by trabeculectomy is required to preserve visual function in XFG [24–27]. In our patients, visual field impairment progressed despite trabeculotomy and lowered IOP, resulting in the need to perform trabeculectomy. Some studies indicated that the results of trabeculectomy in XFG patients were inferior to those in POAG patients, while other studies showed that the results were comparable but more complications occurred [28, 29]. Trabeculectomy is associated with a large number of complications, and the surgical procedure itself, including postoperative management, is burdensome for both surgeons and patients [30, 31]. A key challenge in glaucoma treatment as a whole is to protect the lifelong visual function of patients, which might be achieved by adopting minimally invasive glaucoma surgery and other methods at earlier stages, thereby avoiding subsequent risky surgical procedures such as filtering and tube-shunting. XFG is a type of glaucoma that is well suited for aqueous outflow tract surgery through the trabeculae, and based on its clinical course and disease mechanism, earlier surgery might be advisable. In the future, further studies should be performed to determine whether glaucoma surgery at an earlier stage truly improves treatment and visual function prognosis in XFG patients.

## 5. Conclusion

The XFG patients were older than the POAG and NTG patients, and the duration of previous treatment before referral was shorter for the patients. Moreover, the XFG patients exhibited higher IOP and the eye drop medication score. A significantly higher proportion of XFG patients required surgical intervention compared with those with other disease types.

## Figures and Tables

**Table 1:** Patient characteristics.

	XFG	NTG	POAG
	*n* = 46 eyes	*n* = 54 eyes	*n* = 85 eyes
Age (years)	75.7 ± 8.3 (62–88)	63.3 ± 12.8 (38–77)^*∗∗*^	65.8 ± 12.5 (44–89)^*∗∗*^
Gender (male/female)	18/18	18/17	32/21
Treatment duration (years)	5.1 ± 3.5 (1–14)	8.9 ± 5.9 (2–23)^*∗∗*^	8.9 ± 6.1 (1–29)^*∗∗*^
Medication score	3.6 ± 1.6 (1–7)	2.3 ± 1.3 (0–4)^*∗∗*^	3.1 ± 1.2 (1–6)^*∗∗*^
Intraocular pressure (mmHg)	21.4 ± 8.7 (10–40)	12.9 ± 2.1 (9–19)^*∗∗*^	18.9 ± 6.9 (10–40)^*∗∗*^
VFI (%)	37.9 ± 26.1 (0–98)	47.0 ± 26.5 (1–88)^*∗∗*^	44.1 ± 22.7 (0–93)^*∗∗*^
MD (dB)	−20.9 ± 0.7 (−32 to −3.3)	−15.2 ± 8.5 (−30.5 to −1.9)^*∗∗*^	−18.8 ± 6.8 (−30.1 to −1.3)^*∗*^

^*∗*^ *p* < 0.05, ^*∗∗*^*p* < 0.01, Tukey–Kramer multiple comparison test. MD, mean deviation; NTG, normal-tension glaucoma; POAG, primary open-angle glaucoma; VFI, visual field index; XFG, exfoliation glaucoma.

**Table 2:** Comparison of patients considered to be indicated for surgery according to glaucoma type.

	XFG	NTG	POAG
Not indicated for surgery (# of eyes)	11	48	57^*∗*^
Indicated for surgery (# of eyes)	34	6^*∗∗*^	28^*∗∗*^
Indicated for surgery (>80 years old) (# of eyes)	14	0	4^*∗*^

^*∗*^ *p* < 0.05, ^*∗∗*^*p* < 0.01, Steel–Dwass multiple comparison test. NTG, normal-tension glaucoma; POAG, primary open-angle glaucoma; XFG, exfoliation glaucoma.

**Table 3:** Comparison of exfoliation glaucoma patients according to the surgical procedure for glaucoma.

	XFG *n* = 34 eyes		NTG *n* = 6 eyes		POAG *n* = 28 eyes	
	Average ± SD	Range	Average ± SD	Range	Average ± SD	Range
Age (years)	73.6 ± 7.9	(62–88)	62.3 ± 9.6	(48–73)^*∗∗*^	67.5 ± 12.5	(44–88)^*∗∗*^
Intraocular pressure (mmHg)	22.3 ± 7.8	(10–40)	14.4 ± 2.8	(10–19)^*∗∗*^	20.6 ± 7.1	(13–40)^*∗*^
Antiglaucoma medication score	3.9 ± 1.8	(1–7)	2.8 ± 0.7	(1–3)^*∗∗*^	3.3 ± 0.9	(2–6)^*∗*^
VFI (%)	52.1 ± 29.4	(0–98)	43.5 ± 20.7	(14–69)^*∗∗*^	40.5 ± 20.3	(0–93)^*∗∗*^
MD (dB)	−16.7 ± 8.8	(−30.6–−3.3)	−17.9 ± 6.3	(−26.5–−10.0)^*∗*^	−19.8 ± 7.1	(−30.1–−5.2)^*∗∗*^

^*∗*^ *p* < 0.05, ^*∗∗*^*p* < 0.01, Tukey–Kramer multiple comparison test. MD, mean deviation; NTG, normal-tension glaucoma; POAG, primary open-angle glaucoma; VFI, visual field index; XFG, exfoliation glaucoma.

**Table 4:** Comparison of the surgical procedure and surgical implementation (yes/no) in exfoliation glaucoma patients.

	T-lec/EXPRESS (including nonsimultaneous Cat) *n* = 19 eyes		T-lot triple (including nonsimultaneous Cat) *n* = 15 eyes		Without surgery (including Cat alone) *n* = 11 eyes	
	Average ± SD	Range	Average ± SD	Range	Average ± SD	Range
Age (years)	73.8 ± 9.4	(62–88)	74.3 ± 6.6	(63–84)	81.5 ± 8.5^*∗*^	(69–89)
Treatment duration (years)	5.3 ± 4.1	(1–14)	5.8 ± 3.0	(1.5–11)	5.6 ± 5.2^*∗*^	(1–14)
Medication score	3.9 ± 1.5	(3–7)	3.3 ± 2.0	(1–7)	3.1 ± 0.8^*∗*^	(1–4)
Intraocular pressure at referral (mmHg)	25.4 ± 7.9	(16–40)	20.0 ± 6.6	(10––35)	15.1 ± 5.4^*∗*^	(11–23)
VFI (%)	30.7 ± 23.9	(0–67)	47.6 ± 26.4	(0–80)	65.3 ± 16.5^*∗*^	(42–80)

T-lec, trabeculectomy; EXPRESS, use of express implants; T-lot triple, trabeculotomy + lens reconstruction; Cat, cataract surgery; VFI, visual field index. ^*∗*^*p* < 0.05, Tukey–Kramer multiple comparison test.

**Table 5:** Mean deviation values for the better eye of surgical patients.

	XFG	NTG	POAG
≥−10 dB	21	4	10
<−10 dB	5	2	13^*∗*^

^*∗*^ *p* < 0.05, Steel–Dwass multiple comparison test. NTG, normal-tension glaucoma; POAG, primary open-angle glaucoma; XFG, exfoliation glaucoma.

## Data Availability

The data used to support the findings of this study are available from the corresponding author upon request.
